# Angiopoietin-Like 3 (ANGPTL3) and Atherosclerosis: Lipid and Non-Lipid Related Effects

**DOI:** 10.3390/jcdd5030039

**Published:** 2018-07-14

**Authors:** Maria Giovanna Lupo, Nicola Ferri

**Affiliations:** Dipartimento di Scienze del Farmaco, Università degli Studi di Padova, Padua 35131, Italy; mariagiovanna.lupo@phd.unipd.it

**Keywords:** ANGPTL3, integrin, lipoprotein lipase, monoclonal antibodies, antisense oligonucleotide, atherosclerosis

## Abstract

Genetic and clinical studies have demonstrated that loss-of-function variants in the angiopoietin-like 3 (ANGPTL3) gene are associated with decreased plasma levels of triglycerides (TGs), low-density lipoprotein cholesterol (LDL-C), and high-density lipoprotein cholesterol (HDL-C), which leads to a significant reduction in cardiovascular risk. For this reason, ANGPTL3 is considered an important new pharmacological target for the treatment of cardiovascular diseases (CVDs) together with more conventional lipid lowering therapies, such as statins and anti proprotein convertase subtilisin/kexin type 9 (PCSK9) monoclonal antibodies. Experimental evidence demonstrates that anti-ANGPTL3 therapies have an important anti-atherosclerotic effect. Results from phase I clinical trials with a monoclonal anti-ANGPTL3 antibody (evinacumab) and anti-sense oligonucleotide (ASO) clearly show a significant lipid lowering effect. In addition, from the analysis of the protein structure of ANGPTL3, it has been hypothesized that, beyond its inhibitory activity on lipoprotein and endothelial lipases, this molecule may have a pro-inflammatory, pro-angiogenic effect and a negative effect on cholesterol efflux, implying additional pro-atherosclerotic properties. In the future, data from phase II clinical trials and additional experimental evidence will help to define the efficacy and the additional anti-atherosclerotic properties of anti-ANGPTL3 therapies beyond the already available lipid lowering therapies.

## 1. Introduction

Since its discovery in 1999 by Conklin and colleagues [1], angiopoietin-like 3 (ANGPTL3) has been considered a very potent modulator of triglyceride (TG), low-density lipoprotein cholesterol (LDL-C), and high-density lipoprotein cholesterol (HDL-C) plasma levels by inhibiting lipoprotein lipase (LPL) [2,3,4] and endotelial lipase (EL) activities [5]. Its effect on lipid metabolism was originally identified in a subgroup of inbred strain KK obese mice (name derived from Kondo Keiichi subsequently renamed KK/San strain) [6], expressing a truncated form of ANGPTL3 that was associated with hyperinsulinemia and hyperglycemia. These data indicate that TG levels are positively correlated with a loss of activity of ANGPTL3 [2].

### 1.1. Genetics of ANGPTL3

From experimental evidence, it was then observed that individuals with loss-of-function (LOF) mutations (Table 1) in the *ANGPTL3* gene were affected by familial combined hypolipidemia (FHBL2) and characterized by very low levels of apolipoprotein B (apoB), apolipoprotein A1 (apoA-1) and their associated lipoproteins -very low-density lipoprotein (VLDL), LDL and HDL respectively compared to non-carriers [7,8,9]. These subjects were protected from cardiovascular events, making ANGPTL3 an important pharmacological target for reducing cardiovascular risk, especially for homozygous familial hypercholesterolemic (HoFH) patients, where statins and mAb anti-proprotein convertase subtilisin/kexin type 9 (PCSK9) are not effective [10]. Indeed, plasma TG levels—of which ANGPTL3 is one of the main modulators—have been recognized as cardiovascular risk factors beyond LDL-C levels [11,12].

Carriers of LOF mutations on the *ANGPTL3* gene are associated with a 34% decrease in cardiovascular events [13], and ANGPTL3 plasma levels are closely associated with arterial wall thickness in human subjects [14]. A decreased expression of ANGPTL3 in apolipoprotein E (apoE)-null mice is protective in the development of atherosclerosis [15]. In addition, emerging evidence supports a possible role of ANGPTL3 in the progression of atherosclerosis through a lipid-independent mechanism [16]. Moreover, gain-of-function (GOF) mutations are associated with high plasma TG levels (Table 1).

### 1.2. Genome-Wide Association Studies (GWAS) and ANGPTL3

Many GWAS focused their attention on those traits that associate with different blood lipid profiles and cardiovascular risk, confirming the central role of ANGPTL3 in lipid metabolism. A list of the lead single nucleotide polymorphisms (SNPs) involving regions nearby ANGPTL3 that co-segregate with lipid and cardiovascular risk biomarkers are listed in Table 2. Oldoni and co-workers focused their attention on the SNPs discovered by Teslovich et al. [22] that are concomitantly associated with plasma TG levels and LDL-C levels. Using a combination of in silico and in vitro investigations, Oldoni et al. were able to identify two SNPs (rs6690733 A and rs10889352 T) that increase the expression of ANGPTL3 by affecting putative binding sites for transcription factors [23]. One of them, rs10889352, has been associated with an increase in chromatin accessibility by transcription factors.

## 2. ANGPTL3 Structure

**Figure 1:**
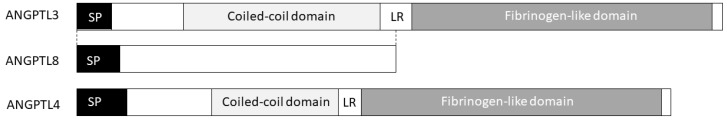
The ANGPTL3/4/8 triad. Belonging to the angiopoietins family, ANGPTL3 is composed of an N-terminal coiled-coil domain involved in LPL (Lipoprotein lipase) and EL (Endothelial lipase) binding and inhibition as well as by a C-terminal fibrinogen-like domain mediating ANGPTL3 angiogenic properties. ANGPTL4 shares with ANGPTL3 both the coiled-coil domain and the fibrinogen-like domain. ANGPTL8 is paralog of the N-terminal region of ANGPTL3 and it is required for ANGPTL3 activation.

ANGPTL3 is a 70 kDa-secreted protein (54 KDa before glycosylation) mainly expressed in the liver, both during embryonic development and in the adult stage [1,17]. It belongs to the angiopoietin growth factor family and shares with its family members an N-teminal α-helix region predicted to fold into a coiled-coil structure and a C-terminal Fibrinogen-like domain (FLD) conserving the overall fold of the fibrinogen domain (~40% of sequence identity), as well as a 16aa signal peptide required for secretion [4,31] (Figure 1). A linker region between N-terminal and C-terminal domains is strictly required for the activation of ANGPTL3 in mice, and its inhibition of LPL activity and the canonical hepatic proprotein convertases (furin, PCSK1, PCSK2, PCSK4, PACE4, PCSK5, and PCSK7) cleave ANGPTL3 into its two domains at the linker level. An N-terminal domain is more efficient at inhibiting LPL activity than the full-length ANGPTL3 [4].

Upon secretion, ANGPTL3 targets the adipose tissue and muscles activating lipolysis in the former, increasing the release of fatty free acids (FFA) and glycerol from adipocytes [32], and inhibiting LPL in the latter, increasing TG-rich lipoproteins (TRLs).

A minor expression of ANGPTL3 is found in healthy kidneys [1], with an increase in its secretion by podocytes in case of renal damage [33,34,35]. To date, the actual role of ANGPTL3 in the kidney is still unknown.

## 3. ANGPTL3 Post-Translational Modifications

ANGPTL3 undergoes several post-translational modifications (Table 3). The GalNAc-T2-mediated O-glycosylation at threonine 226 (T226) plays a crucial role in the accessibility of the nearby cleavage site by proprotein convertases (PCs) [36]. PCs recognize and cleave ANGPTL3 at the RAPR^224^↓TT motif into the linker region (LR) producing an N-terminal-cleaved coiled-coil domain (CCD) that more efficiently inhibits LPL [4]. The O-glycosylation at T226 hinders the cleavage by PCs at arginine 224 (R224). As a consequence, the unprocessed ANGPTL3 is still able to inhibit LPL, but only to a minor extent [36]. GalNAc-T2, encoded by *GALNT2*, is a liver-expressed N-acetylgalactosaminyltransferase that catalyzes the initial step in the pathway of protein glycosylation. It is worth noting that the same GWAS that shed light on the association between *ANGPTL3* and TG, LDL-C and HDL-C also identified *GALNT2* as being correlated with the same phenotypical traits [22,24,29]. Thus, O-glycosylation at T226 GalNAc-T2 significantly affects the activity of ANGPTL3, and thus the plasma TG levels.

## 4. ANGPTL3 Transcriptional Regulation

At transcriptional levels, *ANGPTL3* is mainly regulated by the Liver X receptors (LXRs) and Hepatocyte Nuclear Factor 1α (HNF1α) pathways.

LXRs play a pivotal role in cholesterol homeostasis by inducing the expression of ATP-binding cassette sub-family A member 1 (ABCA1)—essential for HDL formation through reverse cholesterol transport (RCT) from peripheral cells, including macrophages in the vessels [39]—and fatty acid metabolism by inducing the transcription of sterol regulatory element-binding protein-1c (SREBP-1c), fatty acid synthase (FAS) and LPL [40]. The *ANGPTL3* promoter contains LXR responsive elements (LXREs) [41]. A high-cholesterol diet induces ANGPTL3 hepatic expression in mice by activating LXRs [41], leading to hypertriglyceridemia, an effect that is not observed in ANGPTL3-null mice [42]. Notably, LXR also directly triggers the expression of LPL [43] and ANGPTL8 [44], suggesting an LXR-mediated regulatory network among these TG-influencing-level players.

The discovery of an HNF1α-mediated expression (previously supposed by Kaplan’s group [41]) was discovered by treating hypothyroid rats with a subcutaneous injection of thyroid hormone (T3). After treatment, *ANGPTL3* gene expression dramatically reduced by 70% when compared to an untreated control group [45]. This reduction is mediated by thyroid hormone receptor β (TRβ) at the transcriptional level, without affecting ANGPTL3 mRNA stability [45]. The promoter region involved in this inhibition contains LXR and HNF1α binding sites, however only mutations on the latter abolished the expression of *ANGPTL3* after T3 stimulation. Moreover, the TRβ seems to act indirectly on the HNF1α pathway, through a mechanism that does not require DNA binding, since its efficacy is preserved in the TRβ-lacking DNA binding domain. Since co-immunoprecipitation experiments fail to detect TRβ:HNF1α complexes, it has been proposed that TRβ could sequester a limiting co-activator or co-repressor recruited by HNF1α on the *ANGPTL3* promoter [46].

Two known negative regulators of ANGPTL3 transcription are insulin and leptin, which is a relevant issue in hypertriglyceridemia and hyperfattyacidemia in diabetic patients [47,48]. Unfortunately, very little is known on the transcription factors involved in this regulation. To date, no investigations about a putative presence of Insulin Responsive Elements (IREs) have been performed. This could be a relevant issue that needs to be addressed since IREs are involved in insulin-mediated genetic inhibition on ANGPTL3.

## 5. ANGPTL3 Coiled-Coil Fold: Its Role in Lipid Metabolism

By injecting KK/San mice with adenoviral ANGPTL3 deletion mutants missing either the C-terminal FLD or the N-terminal coiled-coil regions, Ono and co-workers highlighted its pivotal role in lipid metabolism [4]. Indeed, the loss of this region prevents the inhibition of LPL and EL by ANGPTL3.

LPL is a lipase anchored to endothelial cells via heparan sulphate-proteoglycans (HSPG) [49,50] and glycerophosphatidylinositol high-density lipoprotein binding protein 1 (GPIHBP1) [51]. It is expressed by skeletal and cardiac muscle, adipose tissue, the lung, the spleen, and lactating mammary glands [52,53]. Upon activation by apolipoprotein C2 (apoC2)-carrying lipoprotein particles (chylomicrons and VLDLs) [54,55], LPL is able to produce ready-to-use TG [56,57]. Since its essential role in the lipid homeostasis it is associated with severe pathological conditions, such as atherosclerosis [58], diabetes, obesity, Alzheimer’s diseases and cachexia [56]. KK/San mice and wild-type mice treated with recombinant ANGPTL3 show a rescue in the low-TG phenotype in the former and hypertriglyceridemia due to ANGPTL3-mediated inhibition of LPL in the latter [2,3,59].

EL is synthesized by endothelial cells and works in the plasma compartment similarly to LPL. It shares an HSPG chain to anchor into luminal endothelial cell membranes. Conversely to LPL, EL acts mainly on HDL fraction, hydrolyzing HDL phospholipids [5,60].

The ANGPTL3 coiled-coil region seems to directly interact with LPL [61] and EL [5]. The ANGPTL3 inhibitory effect is completely abolished in HSPG-missing EL, suggesting a pivotal role of this membrane-anchoring segment in ANGPTL3-induced inhibition of EL activity [5]. Moreover, if ANGPTL3 misses the coiled-coil region, it is not able to inhibit LPL activity [4]. Liu and collaborators suggested that ANGPTL3 could inhibit LPL activity by enhancing its cleavage by the proprotein convertases PACE4 and furin, an effect specific to LPL but not to EL [62]. However, the ANGPTL3 action seems not to be sufficient to inhibit LPL activity, but requires the support of two other angiopoietin-like proteins, namely ANGPTL4 and ANGPTL8.

## 6. The ANGPTL 3-4-8 Model

The discovery of ANGPTL4 as a potent LPL inhibitor precedes that of ANGPTL3 [63,64]. Moreover, bioinformatic searching on the ANGPTL4 LPL binding led to the discovery that ANGPTL3 is the only member of the angiopoietin family that shares a high percentage of both identity and similarity with the LPL binding core of ANGPTL4 [61]. ANGPTL4 levels increase during fasting. LOF mutations on ANGPTL4 develop into a hypolipidemic phenotype [65,66].

ANGPTL8 entered the “wall of fame” of lipid metabolism regulators in 2012 [67,68,69]. It is a feeding-induced hepatokine, highly enriched in the liver, white adipose tissue (WAT) and brown adipose tissue (BAT) [67,68,69], whose LOF mutations cause a low TG condition [69,70]. Indeed, murine animal models in which ANGPTL8 is over-expressed in the liver show a dramatic increase in serum TG levels [68]. Moreover, this increase strictly depends on ANGPTL3 levels, at least in mice [69].

It has been suggested that ANGPTL3-4-8 regulate TG trafficking by inhibiting LPL in different tissues and under different nutritional conditions. While ANGPTL4 and ANGPTL8 levels are strictly dependent on nutritional state in an opposite fashion, ANGPTL3 levels are stable (i.e., its level does not depend on nutritional state), but it requires ANGPTL8 to be activated [69]. Evidence from the work by Quagliarini et al. [69] suggest that ANGPTL8 could itself promote the cleavage at the linker region level and the activation of ANGPTL3, but the authors do not exclude any other mechanism of action.

During fasting conditions or during physical exercise, ANGPTL4 reaches its maximal plasma level, while ANGPTL8 is not synthesized. Under these conditions, ANGPTL4 binds to LPL in WAT, inhibiting its TG hydrolyzing activity [71]. On the other hand, in cardiac and skeletal muscles, under the same conditions, LPL is free from any ANGPTL protein, being free to exert its function. In this way, according to this model, TG are mobilized from WAT to highly oxidizing tissues.

After a defined meal, ANGPTL8 plasma levels increase and ANGPTL4 levels dramatically fall down. ANGPTL8 binds to ANGPTL3 to form a heterodimer which then binds and inhibits LPL in cardiac and skeletal muscles. Conversely, LPL in WAT is free from any inhibition and is able to produce free TG.

So far, there is no evidence that ANGPTL3 and ANGPTL4 act jointly to inhibit LPL. Moreover, ANGPTL4 seems to work alone and not in conjunction with ANGPTL8 or any other ANGPTL proteins. How the ANGPTL3/8 working model fits with the reported ANGPTL3-induced PACE4 and furin in inhibiting LPL activity [62] is still to be fully elucidated.

## 7. ANGPTL3 C-Terminal Domain: Fibrinogen-Like Domain Binds to Integrin αVβ3

Apart from being a pro-atherogenic protein due to its N-terminal coiled-coil LPL/EL-binding domain, ANGPTL3 is deemed to play another role by affecting the arterial thickness [14] and macrophage activity in the lesions through the C-terminal FLD. Conversely to the FLDs from other angiopoietins that bind to endothelial cells via Tie receptors (Tie1 and Tie2) [72], ANGPTL3’s FLD binds to integrin α_V_β_3_ [31]; while this interaction has a proven role in the case of renal damage [33,34,35], a more puzzling scenario is depicted for its importance in atherosclerosis.

Integrin α_V_β_3_ is indeed strongly involved in atherosclerotic plaque formation. Hoshiga et al. showed that α_V_β_3_ correlates with *vasa vasorum* and derived intraplaque vessels both at the endothelial and the smooth muscle cell (SMC) levels. This suggests that SMCs could be stimulated to migrate toward and to accumulate in the intima by several α_V_β_3_ ligands, such as osteopontin [73]. The finding by Camenish and collaborators that ANGPTL3 promotes angiogenesis by binding α_V_β_3_ [31] highlighted once more the importance of ANGPTL3 in atherosclerosis, in which neo-angiogenesis is one of the main hallmarks [74]. Antonov and co-workers described a connection between α_V_β_3_ and foam cell formation during the progression of atherosclerotic lesions [75]. Indeed, macrophages in the lesioned arteries express α_V_β_3_, which suppresses scavenger receptor A (SRA) and CD36 expression, and thus potentially foam cell formation. Moreover, the role of α_V_β_3_ in inflammatory responses is well-known [76], another event that strongly marks the atherosclerotic environment. Thus, the investigation of the possible involvement of ANGPTL3 in inflammation, through the interaction of FLD to α_V_β_3_, could envision new pathophysiological functions.

## 8. Pharmacological Inhibition of ANGPTL3

On July 2017, three different works regarding the pharmacological inhibition of ANGPTL3 have been concomitantly published in the New England Journal of Medicine [77,78,79]. The first two regarded a pre-clinical trial on mice and a phase I clinical trial of evinacumab, a full human monoclonal antibody against ANGPTL3, and the third regarded an inhibition of ANGPTL3 based on an antisense oligonucleotide (ASO) mechanism, tested both in a pre-clinical trial on mice and in a phase I clinical trial.

Evinacumab is able to bind to ANGPTL3 with high affinity and specificity, and to completely reverse its inhibitory activity on LPL and EL both in vitro and in vivo [80]. In vivo studies on normolipidemic C57BL/6 mice showed a dose-dependent reduction in TG, TC, LDL-C and HDL-C serum levels after subcutaneous injections of evinacumab. An increase in LPL and EL activity has been recorded in normolipidemic C57BL/6 mice as well as in dyslipidemic C57BL/6 and *db*/*db* mice [80]. The same results have been obtained by treating dyslipidemic cynomolgus monkeys [80].

Dewey and co-workers showed a consistent lipid-lowering effect in APO*3Leiden—an established strain resembling some features of hyperlipidemic and atherosclerotic patients [81]—as well as healthy mildly-dyslipidemic volunteers with evinacumab (phase I clinical trial). Mice under evinacumab treatment showed a significant decrease in TC and TG content as well as in atherosclerotic lesion size. The phase I trial on healthy volunteers strengthened these results, with a significant reduction of TG, LDL-C and HDL-C levels. The administration of evinacumab to HoFH patients resulted in a nearly 50% reduction of LDL-C levels, along with a similar reduction in apoB and TG levels, as well as HDL-C (−40%) [78].

Graham and collaborators blocked ANGPTL3 action by hampering its translation through an ASO targeting ANGPTL3 mRNA [79]. This ASO has been administered to mice with different lipid backgrounds (wild-type C57BL/6, LDLR knockout, double knockout *ApoC3*^−/−^ *Ldlr*^−/−^, heterozygous *ApoC3*^+/−^ *Ldlr*^−/−^, diet-induce obese mice, mice over-expressing human apoC-III) and to healthy volunteers. A significant decrease in levels of TG, LDL-C and HDL-C in each tested murine strain has been observed, reinforcing the hypothesis that ANGPTL3 lowers LDL-C through a mechanism independent from LDLR. Together with a lipid lowering effect, ASO reduced atherosclerotic plaque development. In this regard, it could be very interesting to see whether ASO can stabilize the plaques in the Tandem Stenosis animal model [82] or reduce the incidence of cardiovascular events in the *ApoE^−/−^Fibrillin-1* animal model [83]. A phase I trial on healthy volunteers confirmed the pre-clinical results, with a significant reduction of TG, LDL-C, HDL-C, apoB and apoC-III [79].

Finally, Chadwick and colleagues [84] exploited a modified CRISPR-Cas9 platform, namely Base Editor 3 (BE3), to permanently inhibit in vivo ANGPTL3 by introducing non-sense mutations within the murine ANGPTL3 gene. This expedient strongly lowers the possibility of undesired *indels* events at double-strand DNA breaks (DSB) sites [85]. After injections with adenoviral vectors expressing BE3-ANGPTL3 in C57BL/6 wild-type mice and in hyperlipidemic *Ldlr*^−/−^ mice, TG and TC levels were halved compared to mice injected with BE3-control. These results are very compelling, paving the way to a complete “non-adherence-to-therapies” troubleshooting method with potentially one lifelong site-specific injection.

## 9. Conclusions

The discovery that LOF mutation of the *ANGPTL3* gene leads to low plasma levels of TGs and cholesterol as well as to a reduction in atherosclerotic lesion size [2,8,14,15,17,31] brought attention to ANGPTL3 as compelling pharmacological target to use, besides the canonical lipid-lowering treatments used in the management of CVDs, such as statins, PCSK9 inhibitors, and ezetimibe. ANGPTL3 inhibitors could result in a complementary support to these treatments, focusing on reducing TG levels as the main objective instead of LDL-C. To date, three strategies for lowering ANGPTL3 have been proposed. Two of them, the full-human monoclonal antibody evinacumab [77,78] and antisense oligonucleotide [79], have proven positive effects on the lipid profile both in pre-clinical trials in murine models and in phase I trial in healthy volunteers and patients affected by homozygous familial hypercholesterolemia. The third strategy is based on an innovative CRISPR/Cas mechanism still under refinement. However, the initial outcomes are encouraging [84].

**Figure 2:**
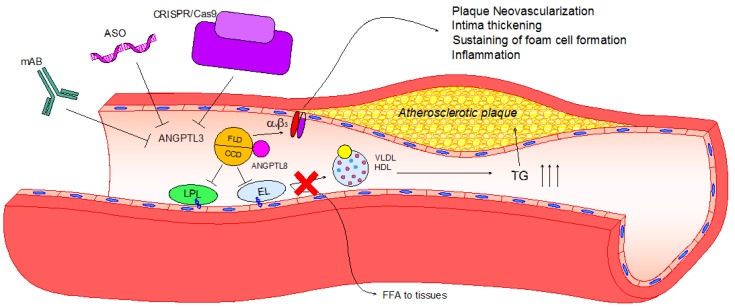
Lipid and non-lipid direct effects of ANGPTL3 and its pharmacological inhibition. Upon activation by ANGPTL8, ANGPTL3 binds to LPL and EL through its coiled-coil domain (CCD), inhibiting their ability to release free fatty acids and phospholipids from VLDL and HDL-C, respectively. Consequently, TG plasma levels increase, eliciting hypertriglyceridemia and atherosclerotic plaque development. Atherosclerotic plaque progression can be enhanced after the activation of the integrin α_V_β_3_ by the fibrinogen-like domain (FLD) of ANGPTL3, leading to plaque neovascularization, intima thickening, foam cell formation and inflammation. To date, three different pharmacological inhibitors have been tested: Monoclonal antibody (evinacumab), antisense oligonucleotide (ASO) and CRISPR/Cas9 editing. All of these effectively reduce ANGPTL3 activity, and thus hypertriglyceridemia and atherosclerotic lesion size in rodent models.

Ignoring the effect of ANGPTL3 on lipid metabolism, the analysis of its peculiar protein structure has suggested additional anti-atherosclerotic effect, such as an anti-inflammatory action, an anti-angiogenic effect, and an increase of macrophage cholesterol efflux. These effects may be mediated through the interaction of ANGPTL3 with the integrin α_V_β_3_, although additional experimental studies are required in order to refine this hypothesis (Figure 2).

Besides the potentially relevant action of ANGPTL3 inhibitors for the treatment of CVDs, the safety of these new therapies is certainly an unresolved issue. Additional and more extensive phase II and III clinical trials are required.

## Figures and Tables

**Table 1:** ANGPTL3 mutations and observed phenotype.

Mutation	Affected Domain	Phenotype	Reference
S17 *	Not CCD N-terminal region	Homozygous carriers: ↓ All lipids (no ANGPTL3 in the plasma) Heterozygous carriers: ↓ Total cholesterol ↓HDL-C (low ANGPTL3 in the plasma)	[7,8]
I19Lfs *	Not CCD N-terminal region	↓ TG ↓ total cholesterol	[17,18]
D41N	Not CCD N-terminal region	↓ TG	[17]
N42D	Not CCD N-terminal region	↓ TG ↓ total cholesterol	[13]
G56V	Not CCD N-terminal region	↓ LDL-C ↓ HDL-C	[19]
F60Lfs *	Not CCD N-terminal region	↓ TG	[13]
K63T	Not CCD N-terminal region	↓ TG (defective LPL inhibition)	[17]
F72L	Not CCD N-terminal region	↓ TG	[17]
T83 *	Not CCD N-terminal region	↓ TG ↓ total cholesterol	[13]
E91G	CCD	↓ TG (defective LPL inhibition)	[17]
E98K	CCD	↓ TG	[17]
N121Kfs	CCD	↓ TG ↓ total cholesterol	[13,20]
S122fs	CCD	↓ TG ↓ total cholesterol	[8,17]
L127F	CCD	↓ TG ↓ LDL-C	[21]
E129 *	CCD	↓ TG ↓ total cholesterol	[7]
K131T	CCD	↓ TG	[17]
N147 *	CCD	↓ TG ↓ total cholesterol	[13,17,18,19]
L164F	CCD	↓ TG (defective LPL inhibition)	[17]
N173S	CCD	↓ TG (defective LPL inhibition)	[17]
Y186 *	CCD	↓ TG ↓ total cholesterol	[13]
Q192 *	CCD	↓ TG ↓ total cholesterol	[13]
S215Lfs *	Linker Region	↓ TG ↓ total cholesterol	[13]
N232fs	Linker Region	↓ TG ↓ total cholesterol	[13]
M259T	FLD	Apparently nonpathogenic	[17]
**R288Q**	**FLD**	**↓ TG (lower ANGPTL3 secretion)**	[13,17]
**S292P**	**FLD**	**↓ TG (lower ANGPTL3 secretion)**	[13,17]
F295L	FLD	↓ LDL-C ↓ HDL-C	[19]
F306Lfs *	FLD	↓ TG ↓ total cholesterol	[13]
R332Q	FLD	↓ LDL-C ↓ HDL-C	[19]
Y347 *	FLD	↓ TG ↓ total cholesterol	[13]
**E375K**	**FLD**	**↓ TG (lower ANGPTL3 secretion)**	[17]
T383S	FLD	↓ TG ↓ total cholesterol	[13]
G400Vfs *	FLD	↓ TG ↓ total cholesterol	[17,18]
W404 *	FLD	↓ TG ↓ total cholesterol	[13]
**Y417C**	**FLD**	**↓ TG (lower ANGPTL3 secretion)**	[17]
A422Qfs *	FLD	↓ TG	[13]
R428M	FLD	↓ TG	[17]
I444Yfs *	FLD	↓ TG	[13]
T454Rfs *	FLD	↓ TG	[13]

CCD: Coiled-coil domain; FLD: Fibrinogen like domain; LPL: lipoprotein lipase; HDL-C: high-density lipoprotein cholesterol; TG: triglycerides; LDL-C: low-density lipoprotein cholesterol. *: Premature stop codon leading to a not functional truncated protein; fs: Frameshift mutation; underlined: L127F missense mutation lowers TG and LDL-C only in Familial Hypercholesterolemia (FH) or Familial Defective apolipoproteinB-100 patients [21]; in bold: These mutations completely abolish or severely decrease the secretion of ANGPTL3 in vitro, suggesting an impairment of the protein fold or stability [17].

**Table 2:** ANGPTL3 Genome-Wide Association Studies lead single nucleotide polymorphisms associated to plasma lipid traits.

SNP ID	Normal Allele	Risk Allele	Phenotypic Trait	Ref.
rs12130333	T	C	TG	[24]
rs10889353	A	C	TG, TC, LDL-C	[25,26,27,28]
rs2131925	T	G	TG, TC, LDL-C	[22,29,30]
rs10889352	C	T	TG, LDL-C	[22,23]
rs6690733	C	A	TG, LDL-C	[22,23]
rs11485618	G	G	LDL-C	[30]
rs995000	C	T	TG	[30]
rs11208004	G	A	TC	[30]

TC: total cholesterol; TG: triglycerides; LDL-C: low-density lipoprotein cholesterol.

**Table 3:** Post-translational modifications (PTM) of ANGPTL3.

PTM	Position(s)	Enzyme	Ref.
N-glycosilation	N115	GlcNAc	[1,37]
**O-glycosilation**	**T226**	**GalNAc-T2**	[36]
Disulfide bond	C246 ↔ C274		[1]
N-glycosilation	N296	GlcNAc	[37,38]
N-glycosilation	N357	GlcNAc	[37]
Disulfide bond	C394 ↔ C408		[1]

In bold are the PTM that preserve ANGPTL3 from PC’s proteolytic cleavage.
